# Prognostic value of inflammatory markers for all-cause mortality in patients with acute myocardial infarction in the coronary care unit: a retrospective study based on MIMIC-IV database

**DOI:** 10.3389/fcvm.2025.1439650

**Published:** 2025-01-22

**Authors:** Fen Cao, Jun-jun Jiang, Gui Zhang, Jun Liu, Ping Xiao, Yang Tian, Wei Zhang, Sheng Zhang, Feng Hou, Zhong-Wu Bao, Kun Wu, Yong-zhi Zhu

**Affiliations:** ^1^ Department of Cardiology, Hunan University of Medicine General Hospital, Huaihua, Hunan, China; ^2^ Department of Neurology, Hunan University of Medicine General Hospital, Huaihua, Hunan, China

**Keywords:** neutrophil-to-lymphocyte ratio, acute myocardial infarction, all-cause mortality, coronary care unit, MIMIC-IV

## Abstract

**Background:**

Acute myocardial infarction (AMI) is prevalent and perilous, leading to mortality and disability in the coronary care unit (CCU). This paper was to verify the correlation of neutrophil-to-lymphocyte ratio (NLR), systemic immune-inflammation index (SII), platelet-to-lymphocyte ratio (PLR), and systemic inflammation response index (SIRI) with all-cause mortality for AMI patients in the CCU.

**Methods:**

Adult patients diagnosed with AMI and admitted to CCU were selected from the MIMIC-IV database. Various clinical and laboratory data were extracted. Logistic regression models were employed to determine the correlation between NLR and in-hospital mortality, 30-day mortality, and 90-day mortality. Confounding factors were adjusted to validate the result robustness. Restricted cubic spline (RCS) curves were adopted to analyze the potential correlation between NLR and all-cause mortality. Meanwhile, the area under the receiver operating characteristic (ROC) curve (AUC) was utilized to compare the prediction ability of NLR, SII, PLR, and SIRI in all-cause mortality. Subsequently, subgroup analyses of gender and comorbidities were performed.

**Results:**

1,386 AMI patients in the CCU were enrolled. The NLR was non-linearly and positively associated with in-hospital mortality [Q4: OR (95%CI) 2.61; (1.261–5.626), *p* = 0.012], 30-day mortality [Q4: OR (95%CI) 2.005; (1.048–3.925); *p* = 0.038], 90-day mortality [Q4: OR (95%CI) 2.191; (1.235–3.948); *p* = 0.008] with Q1 as the reference group. The NLR had the highest AUC for in-hospital mortality, 30-day mortality, and 90-day mortality among four inflammatory markers (NLR, SII, PLR, SIRI). Stratified analyses based on gender and comorbidities showed that the risk of death was significantly increased in male and female patients, with or without diabetes, without cerebral infarction, chronic obstructive pulmonary disease, liver disease, and renal disease in the Q4 group when compared to the Q1 group.

**Conclusions:**

NLR is nonlinearly and positively associated with all-cause mortality of AMI patients in the CCU. The predictive ability of NLR in in-hospital mortality, 30-day mortality, and 90-day mortality is superior to that of SII, PLR, and SIRI.

## Introduction

Acute myocardial infarction (AMI), a prevalent form of coronary heart disease, poses a serious threat to health and life because of its high morbidity and mortality. Previous research has indicated that the in-hospital mortality rate in AMI patients is approximately 10% (1) and the mortality rate for AMI patients in the coronary care unit (CCU) drops to 7% because of professional care (2). Due to high mortality of AMI in the CCU, accurate risk stratification is of great significance for early identification of high-risk individuals and timely adjustment of treatment strategies. Although the GRACE score is commonly used to assess in-hospital mortality from AMI (3), it is limited due to its susceptibility to certain factors and its complicated nature. Risk stratification of AMI patients remains a challenge, primarily due to the lack of standardized prognostic biomarkers in this population. Thereby, it is imperative to explore an easily obtainable and effective index to predict all-cause mortality in AMI patients in the CCU.

The etiology of AMI is intricate and involves multiple factors. Atherosclerosis stands as the primary cardiovascular risk factor for AMI. Inflammatory responses are essential in the initiation and progression of the atherosclerotic process (4–7). The interaction between lymphocytes, neutrophils, monocytes, and platelets could induce inflammation. Ji et al. reported that the neutrophil-to-lymphocyte ratio (NLR) >5.509 was positively correlated with in-hospital mortality in senile AMI patients (8). Chen et al. concluded that systemic immune-inflammation index (SII) and systemic inflammation response index (SIRI) exhibited an independent predictive power for the risk of in-hospital death in senile AMI patients (9). SII and SIRI could be utilized as independent variables for gauging the severity of coronary artery disease (CAD) (10). Li et al. suggested that the platelet-to-lymphocyte ratio (PLR) was connected with in-hospital death risk in AMI individuals (11). Other studies identified SII, PLR, and NLR as strong predictors of mortality in acute coronary syndrome (ACS), and the predictive power of SII was greater than that of PLR and NLR (12). In addition, NLR combined with PLR could better predict in-hospital death risk in AMI patients (13, 14). Surprisingly, a retrospective cohort study stated that NLR could not predict mortality in young AMI patients (15).

Although numerous articles have found the connection between inflammatory markers (NLR, SII, PLR, SIRI) and mortality in AMI individuals, it remains controversial, and there is no evidence of the link between inflammatory markers and all-cause mortality in AMI patients in the CCU. Thereby, this paper delved into the link between NLR and all-cause mortality of AMI patients admitted to CCU, and to evaluate which marker has the best predictive value for all-cause mortality.

## Methods

### Database

This retrospective study was conducted based on the MIMIC-IV database 2.2, a publicly accessible and comprehensive database developed by the MIT Computational Physiology Laboratory. It encompasses medical records of all patients from 2008 to 2019 in the intensive care unit at the Beth Israel Deaconess Medical Center. The first author of this study, Fen Cao, obtained access to the database by completing the Collaborative Institutional Training Initiative course and passing the exams (16). To preserve patient privacy, personal information was anonymous. Therefore, informed consent and ethical approval are not necessary. This study followed the Declaration of Helsinki.

### Study population

**Figure 1:**
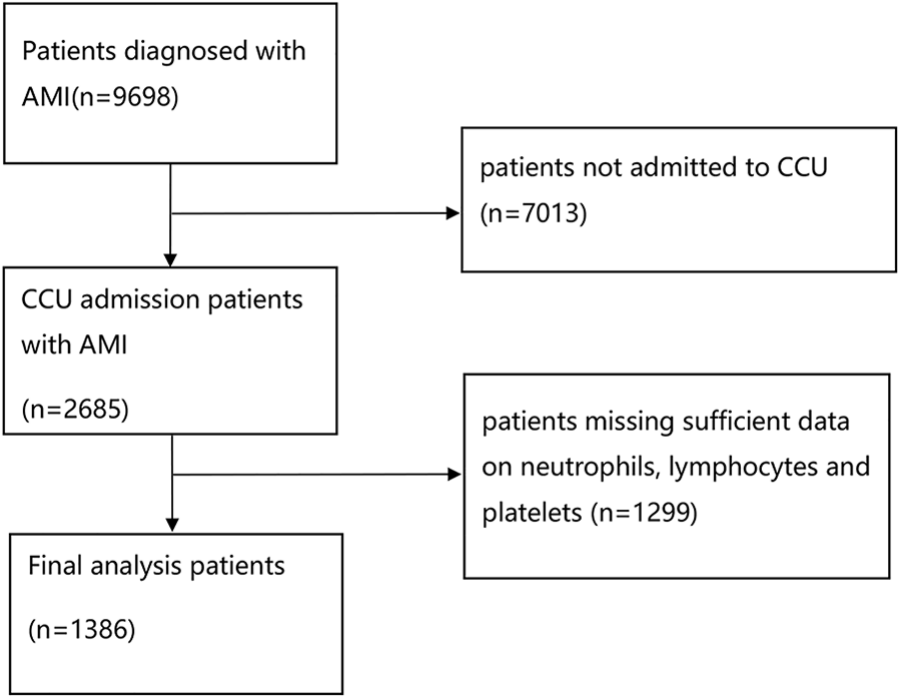
Flowchart of the study patients.

All adult AMI patients in the CCU were diagnosed based on the International Classification of Diseases, 9th and 10th revision. For patients repeatedly admitted to the CCU, only the first CCU admission records were enrolled. Patients were excluded if they were: (1) not admitted to CCU. (2) aged <18 years. (3) without sufficient data on neutrophils, lymphocytes, and platelets within the first CCU admission. Ultimately, 1,386 patients were enrolled (Figure 1).

### Data extraction

The data were extracted using Structured Query Language (SQL). The variables obtained from PostgresSQL (version 16) can be grouped into the following six types: (1) demographic characteristics: admission age, gender, race, BMI, and Charlson comorbidity index (CCI); (2) vital signs: systolic blood pressure (SBP), diastolic blood pressure (DBP), oxygen saturation, and temperature. (3) laboratory parameters: hemoglobin, hemoglobina1c, total bilirubin, INR, BUN, creatinine, platelet count, lymphocyte count, neutrophil count, and monocyte count. (4) drug therapy: aspirin, clopidogrel, heparin, tirofiban, statin, esmolol, amiodarone, digoxin, and milrinone. We also investigated whether patients underwent PCI or coronary artery bypass grafting (CABG) during hospitalization. (5) comorbidities: diabetes, cerebral infarction, chronic obstructive pulmonary disease (COPD), liver disease, and renal disease. (6) scoring systems: acute physiology score III (APS III), oxford acute severity of illness score (OASIS), systemic inflammatory response syndrome score (SIRS), simplified acute physiology score II (SAPS II, Glasgow coma scale (GCS), logistic organ dysfunction system (LODS). For variables measured several times, only the first value was obtained. To reduce the bias from sample exclusion, the percentage of missing values for each continuous variable was calculated. For variables with a proportion of missing value <30%, the multiple interpolation method was utilized to predict the missing value five times, and the mean value was computed as the final result. Interfering variables were deleted via clinical expertise in case of over 30% of missing values.

### Definition

NLR was computed as neutrophil/lymphocyte. SII was computed as platelet ×neutrophil/lymphocyte. PLR was computed as platelet/lymphocyte. SIRI was computed as neutrophil × monocyte/lymphocyte. (All individuals were grouped based on NLR quartiles, with Q1 as the reference). The primary endpoint was all-cause mortality of AMI patients in the CCU (in-hospital mortality), and the secondary endpoints were 30-day all-cause mortality (30-day mortality) and 90-day all-cause mortality (90-day mortality).

### Statistical analysis

Continuous variables in normal distribution were depicted as mean and standard deviation (SD), and differences were estimated using the *t*-test, while variables in abnormal distribution were reported as the median and interquartile range (IQR) and estimated by the Kruskal-Wallis test. Besides, categorical variables were reported as frequency and percentage, and differences were estimated by the Chi^2^ test. Logistic regression models were adopted to judge the associations between NLR and both the primary and secondary endpoints. Different models were utilized to adjust for potential confounding variables. Once NLR was grouped based on quartiles, with Q1 as the reference group, the adjusted odds ratio (OR) and 95% confidence interval (CI) were computed to judge the correlation between NLR and different endpoints. Model 1 was unadjusted, while Model 2 was adjusted for demographic features, vital signs, and laboratory parameters, including admission age, gender, race, BMI, CCI, SBP, DBP, oxygen saturation, temperature, hemoglobin, hemoglobina1c, total bilirubin, INR, BUN, creatinine, and monocyte count. Model 3 was additionally adjusted for clinical therapy, comorbidities, and scoring systems, including aspirin, clopidogrel, heparin, tirofiban, statin, esmolol, amiodarone, digoxin, milrinone, PCI, CABG, diabetes, cerebral infarction, COPD, liver disease, renal disease, APS III, OASIS, SIRS, SAPS II, GCS, and LODS based on Model 2. Additionally, when NLR was used as a continuous variable, restricted cubic spline (RCS) curves were employed to further investigate the link between NLR and all-cause mortality. Furthermore, the receiver operating characteristic (ROC) curves of NLR, SII, PLR, and SIRI were plotted for predicting in-hospital, 30-day, and 90-day mortality in AMI individuals, and the predictive value was compared via the area under the ROC curve (AUC). Finally, subgroup analyses were implemented to elucidate the relationship between NLR and all-cause mortality in various subgroups, encompassing sex and comorbidities (diabetes, cerebral infarction, COPD, liver disease, and renal disease). The integrated discrimination improvement (IDI) was calculated to assess the improvement in the predictive power and clinical value of scoring tools resulting from the NLR. Statistical analyses were done using the software package R (version 4.3.2), and *p* < 0.05 from two-tailed tests implied statistical significance.

## Results

### Baseline characteristics

1,386 CCU patients who suffered from AMI were included, with an in-hospital mortality of 11.04% (153 died), a 30-day mortality of 12.63% (175 died), and a 90-day mortality of 16.67% (231 died). According to the NLR quartiles, the patients were allocated equally into four groups. The median (IQR) admission age was 71.53 (62.70–80.66), 59.7% of patients were males, and 63.9% of patients were whites. The median (IQR) BMI was 28.40 (24.60–33.20). On admission, there were differences in terms of CCI, DBP, oxygen saturation, hemoglobin, total bilirubin, INR, BUN, creatinine, monocyte count, aspirin, clopidogrel, statin, amiodarone, CABG, APS III, OASIS, SIRS, SAPS II, GCS, and LODS between the high NLR group and the low NLR group. Baseline characteristics are depicted in Table 1. Baseline information grouped by in-hospital mortality is shown in Supplementary Table 1.

**Table 1:** Baseline characteristics of patients.

Characteristic	Total (*n* = 1,386)	Q1 (*n* = 347)	Q2 (*n* = 346)	Q3 (*n* = 346)	Q4 (*n* = 347)	*p*
Admission age (years)	71.53 [62.70, 80.66]	70.45 [59.34, 79.57]	70.96 [61.79, 79.42]	72.33 [65.11, 82.24]	72.45 [64.94, 81.08]	0.003
Gender (male)	827 (59.7%)	187 (53.9%)	208 (60.1%)	214 (61.8%)	218 (62.8%)	0.073
Race (other)	300 (21.6%)	113 (32.6%)	71 (20.5%)	65 (18.8%)	51 (14.7%)	<0.001
Race (white)	885 (63.9%)	205 (59.1%)	232 (67.1%)	238 (68.8%)	210 (60.5%)	
Race (unknow)	201 (14.5%)	29 (8.4%)	43 (12.4%)	43 (12.4%)	86 (24.8%)	
BMI (kg/m^2^)	28.40 [24.60, 33.20]	29.00 [25.35, 33.45]	28.90 [24.30, 33.20]	28.55 [24.70, 33.30]	27.60 [24.20, 32.40]	0.053
CCI	7.00 [5.00, 9.00]	7.00 [5.00, 9.00]	7.00 [5.00, 9.00]	8.00 [6.00, 10.00]	8.00 [6.00, 10.00]	0.028
Vital signs						
SBP (mmHg)	128.00 [117.00, 142.00]	127.00 [118.00, 140.00]	128.00 [116.25, 142.00]	129.00 [114.25, 140.00]	130.00 [117.00, 143.00]	0.912
DBP (mmHg)	71.00 [62.00, 80.00]	74.00 [65.00, 80.00]	70.00 [61.00, 80.00]	70.00 [60.00, 80.00]	70.00 [62.00, 80.00]	0.004
Oxygen saturation (%)	97.00 [95.00, 99.00]	98.00 [95.00, 100.00]	97.00 [95.00, 99.00]	97.00 [95.00, 99.00]	97.00 [94.00, 100.00]	0.014
Temperature (℃)	36.67 [36.44, 36.89]	36.61 [36.44, 36.89]	36.67 [36.44, 36.93]	36.67 [36.44, 36.89]	36.67 [36.44, 36.94]	0.993
Laboratory parameters						
Hemoglobin (g/dl)	12.70 [11.10, 14.07]	13.10 [11.50, 14.30]	13.00 [11.43, 14.30]	12.30 [11.00, 13.80]	12.20 [10.40, 13.80]	<0.001
Hemoglobina1c (%)	6.00 [5.60, 7.00]	6.10 [5.60, 7.00]	6.00 [5.60, 7.10]	6.10 [5.60, 7.10]	6.00 [5.50, 6.90]	0.266
Total bilirubin (mg/dl)	0.50 [0.30, 0.80]	0.50 [0.30, 0.70]	0.50 [0.30, 0.80]	0.50 [0.40, 0.70]	0.60 [0.40, 0.80]	0.007
INR	1.10 [1.00, 1.30]	1.10 [1.00, 1.20]	1.10 [1.00, 1.20]	1.10 [1.00, 1.20]	1.20 [1.10, 1.40]	<0.001
BUN (mmol/L)	21.00 [16.00, 30.00]	18.00 [14.00, 24.00]	21.00 [16.00, 27.00]	21.00 [16.00, 31.00]	25.00 [17.00, 36.00]	<0.001
Creatinine (mg/dl)	1.10 [0.90, 1.40]	0.90 [0.80, 1.20]	1.00 [0.80, 1.40]	1.10 [0.90, 1.50]	1.20 [0.90, 1.70]	<0.001
Platelet count(10^9^/L)	228.00 [179.00, 282.00]	238.00 [188.00, 297.00]	225.00 [177.50, 272.50]	223.00 [180.50, 281.75]	222.00 [174.00, 280.00]	0.019
Lymphocyte count(10^9^/L)	1.35 [0.89, 1.98]	2.14 [1.67, 2.73]	1.56 [1.25, 2.04]	1.20 [0.91, 1.56]	0.71 [0.49, 1.00]	<0.001
Neutrophil count(10^9^/L)	6.86 [4.73, 10.44]	4.09 [3.23, 5.31]	6.15 [4.82, 7.55]	8.23 [6.30, 10.60]	12.56 [9.64, 15.35]	<0.001
Monocyte count(10^9^/L)	0.72 [0.52, 0.97]	0.65 [0.51, 0.86]	0.68 [0.52, 0.89]	0.76 [0.56, 1.02]	0.81 [0.49, 1.20]	<0.001
Clinical therapy, *n* (%)						
Aspirin	1,327 (95.7%)	341 (98.3%)	330 (95.4%)	335 (96.8%)	321 (92.5%)	0.001
Clopidogrel	845 (61.0%)	224 (64.6%)	199 (57.5%)	225 (65.0%)	197 (56.8%)	0.036
Heparin	1,355 (97.8%)	342 (98.6%)	342 (98.8%)	336 (97.1%)	335 (96.5%)	0.117
Tirofiban	73 (5.3%)	26 (7.5%)	19 (5.5%)	12 (3.5%)	16 (4.6%)	0.11
Statin	1,303 (94.0%)	331 (95.4%)	323 (93.4%)	333 (96.2%)	316 (91.1%)	0.02
Esmolol	39 (2.8%)	12 (3.5%)	10 (2.9%)	6 (1.7%)	11 (3.2%)	0.538
Amiodarone	410 (29.6%)	90 (25.9%)	93 (26.9%)	105 (30.3%)	122 (35.2%)	0.033
Digoxin	128 (9.2%)	22 (6.3%)	37 (10.7%)	33 (9.5%)	36 (10.4%)	0.178
Milrinone	87 (6.3%)	16 (4.6%)	25 (7.2%)	25 (7.2%)	21 (6.1%)	0.436
PCI	285 (20.6%)	93 (26.8%)	88 (25.4%)	60 (17.3%)	44 (12.7%)	<0.001
CABG	243 (17.5%)	53 (15.3%)	76 (22.0%)	68 (19.7%)	46 (13.3%)	0.01
Comorbidities, *n* (%)						
Diabetes	691 (49.9%)	178 (51.3%)	168 (48.6%)	186 (53.8%)	159 (45.8%)	0.18
Cerebral infarction	139 (10.0%)	44 (12.7%)	32 (9.2%)	27 (7.8%)	36 (10.4%)	0.181
COPD	275 (19.8%)	64 (18.4%)	70 (20.2%)	63 (18.2%)	78 (22.5%)	0.465
Liver disease	74 (5.3%)	22 (6.3%)	22 (6.4%)	14 (4.0%)	16 (4.6%)	0.406
Renal disease	152 (11.0%)	26 (7.5%)	38 (11.0%)	43 (12.4%)	45 (13.0%)	0.091
Scoring systems						
APS III	48.00 [35.00, 63.00]	44.00 [30.00, 60.50]	46.00 [32.00, 58.00]	48.00 [35.25, 64.75]	54.00 [40.00, 65.50]	<0.001
OASIS	33.00 [27.00, 40.00]	32.00 [25.00, 38.00]	32.50 [26.00, 39.00]	32.00 [26.00, 40.00]	36.00 [30.00, 42.00]	<0.001
SIRS	3.00 [2.00, 3.00]	3.00 [2.00, 3.00]	3.00 [2.00, 3.00]	3.00 [2.00, 3.00]	3.00 [2.00, 3.00]	<0.001
SAPS II	40.00 [31.00, 51.00]	37.00 [28.00, 49.00]	38.00 [29.00, 48.00]	41.00 [31.00, 51.00]	45.00 [35.50, 54.00]	<0.001
GCS	14.00 [11.00, 15.00]	14.00 [11.50, 15.00]	14.00 [12.00, 15.00]	14.00 [11.00, 15.00]	14.00 [10.00, 15.00]	0.032
LODS	3.00 [1.00, 5.00]	2.00 [1.00, 4.00]	3.00 [1.00, 4.00]	3.00 [1.00, 5.00]	4.00 [2.00, 7.00]	<0.001

BMI, body mass index; CCI, Charlson comorbidity index; SBP, systolic blood pressure; DBP, diastolic blood pressure; INR, international normalized ratio; BUN, blood urea nitrogen; PCI, percutaneous coronary intervention; CABG, coronary artery bypass grafting; COPD, chronic obstructive pulmonary disease; APS III, acute physiology score III; OASIS, Oxford acute severity of illness score; SIRS, systemic inflammatory response syndrome score; SAPS II, simplified acute physiology score II; GCS, Glasgow coma scale; LODS, logistic organ dysfunction system.

### Association between NLR and all-cause mortality in AMI patients

**Figure 2:**
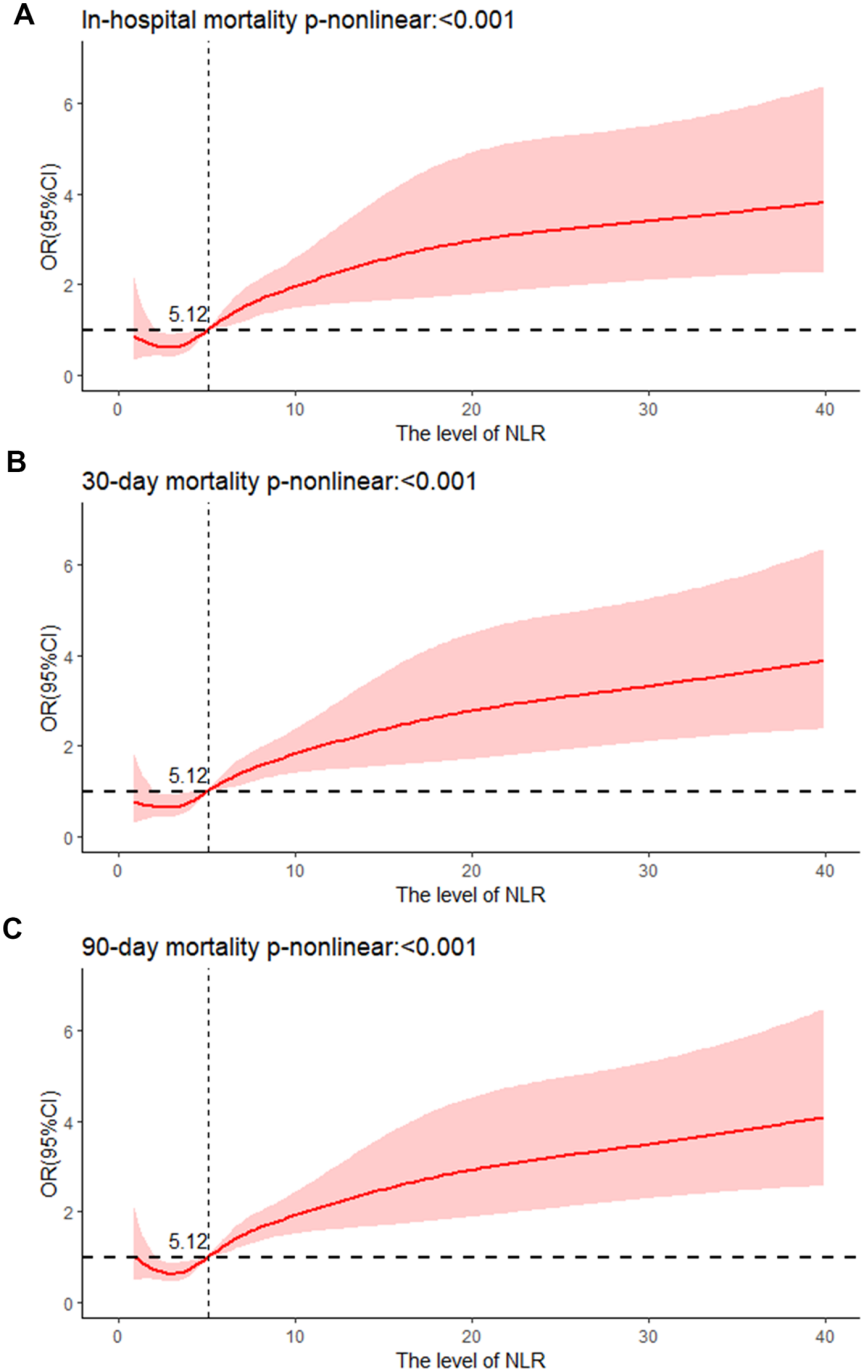
Cubic spline plot of relation of NLR to **(A)** risk of in-hospital mortality, cubic spline plots of NLR in relation to **(B)** risk of 30-day mortality, and **(C)** risk of 90-day mortality.

According to the unadjusted Logistic regression model (model 1), in-hospital [Q4: OR; (95%CI) 4.356; (2.638–7.504)], 30-day [Q4: OR; (95%CI) 3.699; (2.339–6.025)], and 90-day mortality rates [Q4: OR; (95%CI) 3.597; (2.395–5.507)] (all *p* < 0.001) were enhanced in Q4 group with Q1 as the reference group. After modifying for demographic features, vital signs, and laboratory parameters (Model 2), in-hospital mortality [Q4: OR; (95%CI) 3.067; (1.754–5.554)], 30-day mortality [Q4: OR (95%CI) 2.521; (1.512–4.304)], and 90-day mortality [Q4: OR (95%CI) 2.721; (1.715–4.387)] (all *p* < 0.001) were also increased in Q4 group in contrast to Q1 group. Based on Model 2, the confounding variables were further adjusted for clinical therapy, comorbidities, and scoring systems (Model 3). NLR was positively correlated with in-hospital [Q4: OR (95%CI) 2.61; (1.261–5.626), *p* = 0.012], 30-day [Q4: OR (95%CI) 2.005; (1.048–3.925); *p* = 0.038], and 90-day mortality rates [Q4: OR (95%CI) 2.191; (1.235–3.948); *p* = 0.008] in contrast to Q1 group (Table 2). Moreover, the RCS revealed that NLR was non-linearly and positively associated with in-hospital, 30-day, and 90-day mortality rates (all *p* < 0.001, and death risk was increased gradually when NLR >5.12 (Figures 2A–C).

**Table 2:** The association of NLR with all-cause mortality.

		Model 1		Model 2		Model 3	
		OR (95%CI)	*p*	OR (95%CI)	*p*	OR (95%CI)	*p*
In-hospital mortality	Q1	ref	–	ref	–	ref	–
	Q2	1.164 (0.627–2.179)	0.63	1.005 (0.524–1.941)	0.988	1.279 (0.562–2.957)	0.559
	Q3	1.958 (1.124–3.506)	0.02	1.451 (0.797–2.701)	0.23	1.685 (0.793–3.704)	0.183
	Q4	4.356 (2.638–7.504)	<0.001	3.067 (1.754–5.554)	<0.001	2.61 (1.261–5.626)	0.012
30-day mortality	Q1	ref	–	ref	–	ref	–
	Q2	0.92 (0.515–1.639)	0.777	0.795 (0.433–1.454)	0.456	0.891 (0.424–1.871)	0.761
	Q3	1.846 (1.119–3.103)	0.018	1.324 (0.771–2.306)	0.314	1.431 (0.738–2.835)	0.294
	Q4	3.699 (2.339–6.025)	<0.001	2.521 (1.512–4.304)	<0.001	2.005 (1.048–3.925)	0.038
90-day mortality	Q1	ref	–	ref	–	ref	–
	Q2	0.911 (0.552–1.499)	0.713	0.788 (0.462–1.340)	0.38	0.85 (0.445–1.620)	0.621
	Q3	1.812 (1.169–2.845)	0.009	1.325 (0.819–2.164)	0.255	1.491 (0.833–2.706)	0.183
	Q4	3.597 (2.395–5.507)	<0.001	2.721 (1.715–4.387)	<0.001	2.191(1.235–3.948)	0.008

Model 1: unadjusted. Model 2: adjusted for demographic features, vital signs and laboratory parameters, including admission age, gender, race, BMI, CCI, SP, DP, oxygen saturation, temperature, hemoglobin, hemoglobina1c, total bilirubin, INR, BUN, creatinine and monocyte count. Model 3 was additionally adjusted for clinical therapy, comorbidities and scoring systems based on Model 2, including aspirin, clopidogrel, heparin, tirofiban, statin, esmolol, amiodarone, digoxin, milrinone, PCI, CABG, diabetes, cerebral infarction, COPD, liver disease, renal disease, APS III, OASIS, SIRS, SAPS II, GCS, LODS.

### Discriminative power of inflammatory markers for predicting all-cause mortality

**Figure 3:**
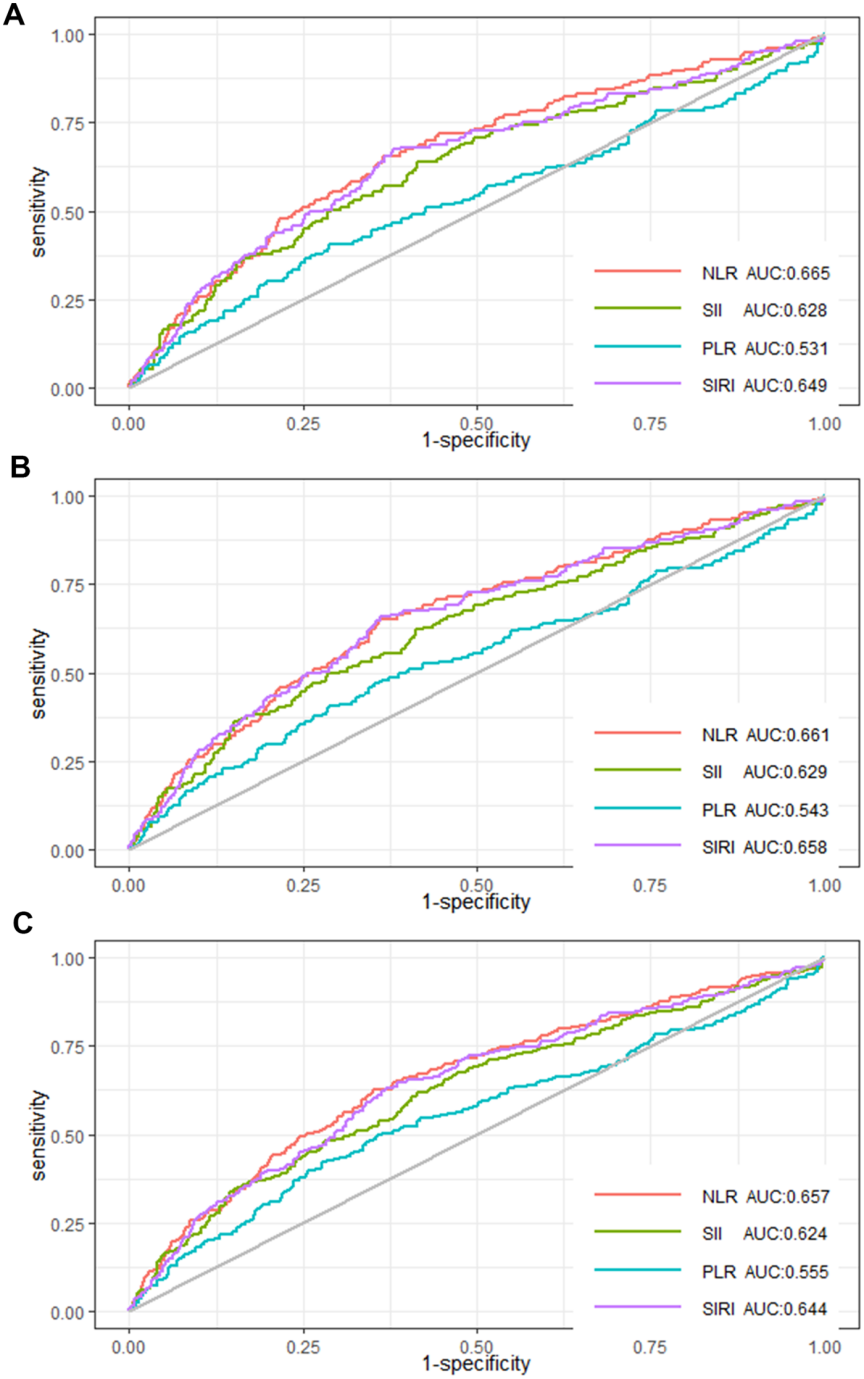
ROC curves of NLR, SII, PLR, and SIRI for **(A)** in-hospital mortality, **(B)** 30-day mortality, and **(C)** 90-day mortality.

ROC curves were used to illustrate the predicting power of the four inflammatory indicators (NLR, SII, PLR, SIRI) for in-hospital, 30-day, and 90-day mortality in AMI patients in the CCU. The AUC of in-hospital mortality predicted by NLR was 0.665, the AUC by SII was 0.628, the AUC by PLR was 0.531 and the AUC by SIRI was 0.649 (Figure 3A). The AUC of 30-day mortality predicted by NLR was 0.661, the AUC by SII was 0.629, the AUC by PLR was 0.543 and the AUC by SIRI was 0.658 (Figure 3B). The AUC of 90-day mortality predicted by NLR was 0.657, the AUC by SII was 0.624, the AUC by PLR was 0.555 and the AUC by SIRI was 0.644 (Figure 3C). These results evinced that NLR has the best power than SII, PLR, and SIRI in predicting in-hospital, 30-day, and 90-day mortality.

### Subgroup analysis

To further confirm the association between NLR and in-hospital, 30-day, and 90-day mortality rates, stratified analyses were implemented based on gender and comorbidities (diabetes, cerebral infarction, COPD, liver disease, and renal disease). There was a significant correlation between NLR and all-cause mortality for both males [Q4: OR; (95%CI) 2.886; (1.683–5.128), *p* < 0.001] and females [Q4: OR; (95%CI) 4.988; (2.708–9.605), *p* < 0.001] in contrast to the Q1 group in the full adjusted model, as well as for patients with diabetes [Q4: OR; (95%CI) 2.813; (1.570–5.196), *p* < 0.001] and those without diabetes [Q4: OR; (95%CI) 4.4; (2.498–8.118), *p* < 0.001] compared to the Q1 group. Subgroup analysis also revealed a notable link between NLR and all-cause mortality in patients without cerebral infarction [Q4: OR; (95%CI) 3.882; (2.507–6.162); *p* < 0.001], COPD[Q4: OR; (95%CI) 3.707; (2.354–5.980); *p* < 0.001], liver disease [Q4: OR; (95%CI) 3.587; (2.373–5.531); *p* < 0.001], renal disease [Q4: OR; (95%CI) 3.636; (2.391–5.636); *p* < 0.001] with Q1 as the reference group. For outcome variables regarding in-hospital, 30-day, and 90-day mortality, subgroup analysis consistently demonstrated a similar relationship of NLR across various subgroups (Table 3).

**Table 3:** Subgroup analysis of gender and comorbidities.

Subgroups	In-hospital mortality		30-day mortality		90-day mortality	
	OR (95%CI)	*p*	OR (95%CI)	*p*	OR (95%CI)	*p*
Gender						
Females	5.767 (2.748–13.31)	<0.001	5.044 (2.516–10.86)	<0.001	4.988 (2.708–9.605)	<0.001
Males	3.596 (1.846–7.570)	<0.001	2.983 (1.637–5.718)	<0.001	2.886 (1.683–5.128)	<0.001
Diabetes						
No	3.452 (1.827–6.932)	<0.001	3.587 (1.937–7.027)	<0.001	4.4 (2.498–8.118)	<0.001
Yes	5.916 (2.666–15.01)	<0.001	3.763 (1.921–7.846)	<0.001	2.813 (1.570–5.196)	<0.001
Cerebral infarction						
No	4.532 (2.648–8.165)	<0.001	3.918 (2.398–6.639)	<0.001	3.882 (2.507–6.162)	<0.001
Yes	3.299 (0.841–16.31)	0.103	2.414 (0.666–9.940)	0.19	2.111 (0.681–6.967)	0.201
COPD						
No	4.791 (2.744–8.824)	<0.001	3.975 (2.390–6.860)	<0.001	3.707 (2.354–5.980)	<0.001
Yes	3 (0.999–11.11)	0.067	2.81 (1.018–9.075)	0.059	3.199 (1.320–8.636)	0.054
Liver disease						
No	4.398 (2.634–7.683)	<0.001	3.7 (2.319–6.086)	<0.001	3.587 (2.373–5.531)	<0.001
Yes	3 (0.263–68.26)	0.388	3 (0.263–68.26)	0.388	3 (0.263–68.26)	0.388
Renal disease						
No	4.445 (2.646–7.802)	<0.001	3.774 (2.352–6.239)	<0.001	3.636 (2.391–5.636)	<0.001
Yes	4.605 (0.754–88.83)	0.165	4.605 (0.754–88.83)	0.165	6.25(1.074–119.0)	0.091

Data were adjusted for admission age, gender, race, BMI, CCI, SP, DP, oxygen saturation, temperature, hemoglobin, hemoglobina1c, total bilirubin, INR, BUN, creatinine, monocyte count, aspirin, clopidogrel, heparin, tirofiban, statin, esmolol, amiodarone, digoxin, milrinone, PCI, CABG, diabetes, cerebral infarction, COPD, liver disease, renal disease, APS III, OASIS, SIRS, SAPS II, GCS, and LODS. The variables examined in this table were not adjusted.

### The incremental effect of the NLR

The IDI of the scoring tools (APSIII, OASIS, SIRS, SAPSII, GCS, LODS) was calculated to analyze the impact of the NLR on the predictive ability to score tools. IDI is a tool to assess the improvement in the predictive ability of the model, with a value greater than 0 indicating a positive improvement and a value less than 0 indicating a negative improvement. The results showed that the predictive ability of the scoring tools with the NLR was improved in comparison to those without the NLR. After considering the NLR according to quartile classification [NLR (IQR)], the predictive ability of the scoring tool (APSIII, OASIS, SIRS, SAPSII, GCS) for all-cause mortality and LODS of 90-day mortality was significantly improved (*P* < 0.05), whereas the improvement in the predictive ability of LODS in in-hospital and 30-day mortality was not statistically significant (*P* > 0.05) (Table 4).

**Table 4:** The incremental effect of the NLR.

	In-hospital mortality		30-day mortality		90-day mortality	*p*
	IDI (95%CI)	*p*	IDI (95%CI)	*p*	IDI (95%CI)	
APS III	0.039 (0.024–0.053)	<0.001	0.037 [0.024–0.050]	<0.001	0.039 (0.027–0.052)	<0.001
OASIS	0.037 [0.023–0.050]	<0.001	0.035 [0.023–0.047]	<0.001	0.039[0.027–0.051]	<0.001
SIRS	0.033 [0.023–0.044]	<0.001	0.035 [0.025–0.045]	<0.001	0.041 [0.030–0.052]	<0.001
SAPS II	0.033 [0.019–0.047]	<0.001	0.032 [0.020–0.045]	<0.001	0.035[0.022–0.047]	<0.001
GCS	0.036 [0.023–0.049]	<0.001	0.036 [0.025–0.048]	<0.001	0.043 [0.031–0.056]	<0.001
LODS	0.004 [−0.005–0.013]	0.386	0.006 [−0.001–0.014]	0.11	0.008 [4e-04–0.015]	0.038

IDI, integrated discrimination improvement; APS III, acute physiology score III; OASIS, Oxford acute severity of illness score; SIRS, systemic inflammatory response syndrome score; SAPS II, simplified acute physiology score II; GCS, Glasgow coma scale; LODS, logistic organ dysfunction system.

## Discussion

This paper demonstrated a positive and nonlinear relationship between NLR and in-hospital, 30-day, and 90-day mortality among AMI individuals in the CCU, and all-cause mortality was increased gradually when NLR >5.12. NLR was better than SII, PLR, and SIRI in forecasting in-hospital, 30-day, and 90-day mortality. Thus, NLR is an excellent inflammatory marker for predicting all-cause mortality for AMI patients in the CCU.

NLR (neutrophil to lymphocyte ratio) is a novel inflammatory marker. Previous research has illustrated that NLR is directly connected with in-hospital all-cause mortality and long-term outcomes in AMI patients. Three meta-analyses with over 10,000 patients each showed that NLR served as an independent predictor for in-hospital mortality and long-term prognosis in individuals with ST-elevation myocardial infarction (STEMI) undergoing PCI (17–19). A retrospective and observational study with 2,618 Chinese AMI patients discovered a positive link between NLR >5.509 and in-hospital death risk. Compared to PLR, NLR had a superior ability in predicting in-hospital death in non-STEMI patients (8). Furthermore, NLR (≥6.07) could predict major adverse cardiovascular events (MACEs) in AMI patients (20). NLR >5.77 could independently predict in-hospital mortality for AMI (21) and NLR was associated with 30-day all-cause mortality, gastrointestinal hemorrhage, MACEs, and non-fatal stroke (22). Meanwhile, NLR is a marked indicator for 1-year reinfarction and mortality in AMI patients complicated with diabetes (23, 24). Besides, NLR ≥3.9 for STEMI and ≥2.7 for NSTEMI patients following PCI within 24-h significantly predicted 1-year cardiovascular mortality (25), and NLR ≥3.39 independently predicted 2-year all-cause death (26). NLR could also strongly predict 3-year mortality in NSTEMI patients (27). Similarly, our study proved that NLR was positively associated with in-hospital, 30-day, and 90-day mortality, and death risk was increased gradually when NLR >5.12. The prediction power of NLR in all-cause mortality outperformed SII, PLR, and SIRI in AMI patients in the CCU. Therefore, clinicians can identify high-risk patients based on this specific threshold of NLR, adopt optimal preventive strategies, and make more aggressive treatment decisions.

Many studies have shown the association of NLR with AMI complications and serious conditions of coronary arteries. Nunez et al. discovered that the NLR could be a pivotal prognostic tool for cardiac shock, a life-threatening AMI complication (28). Left ventricular thrombosis (LVT) is another devastating AMI complication. Studies have indicated the association between inflammatory biomarkers and LVT development (29). AMI patients who had LVT and did not receive PCI exhibited stronger inflammatory responses and higher levels of NLR and PLR. Hence, LVT did not dissolve in these patients despite anticoagulation therapy (30). Besides, an elevated NLR was related to in-hospital malignant ventricular arrhythmia after PCI in STEMI patients (31) and also predicted left ventricular systolic dysfunction in patients with NST-ACS (32). Moreover, an elevated NLR was linked to coronary slow flow and no-reflow in AMI patients (33, 34). During the long-term follow-up of NSTEMI patients with coronary slow flow, NLR >3.88 independently predicted recurrent AMI (35). Admission NLR is also associated with SYNTAX score in AMI patients treated with PCI (25). Multiple studies concluded the positive correlation between NLR and GRACE (36) and the severity of coronary artery lesions in AMI patients (37). Overall, elevated NLR was closely connected with MACEs, AMI complications, serious conditions of coronary arteries, gastrointestinal hemorrhage, and stroke, thereby increasing all-cause mortality in AMI patients.

The pathophysiological process of AMI is complicated, including atherosclerosis, plaque rupture, and thrombosis. Inflammation is commonly utilized as a risk-stratified indicator that forecasts adverse events and is key in advanced atherosclerosis with the involvement of immune cells, such as neutrophils, lymphocytes, monocytes, and platelets (38–40). Neutrophils, the predominant type of leukocytes in peripheral blood, exert crucial roles in inflammation. These cells are known to promote smooth muscle cell lysis and apoptosis and then promote inflammation during atherosclerosis (41). Physical or biochemical damage to the coronary activates and recruits platelets and in turn stimulates the atherosclerotic process through the interaction of leukocytes, endothelial cells, and inactivated platelets (42). Neutrophils assemble in endothelial injury sites through chemokines, cytokines, and adhesion molecules, and interact with platelets to enhance monocyte infiltration into the injured endothelium, leading to atherosclerosis. Neutrophils also promote atherosclerotic plaque rupture by releasing cytokines and reactive oxygen species that activate macrophage foam cells by producing oxidized lipids (43). Studies have found that low lymphocyte count is associated with atherosclerosis, inflammation, and endothelial functions in AMI patients (44). The more neutrophils, the fewer lymphocytes, the higher inflammation and stress levels, and the more serious the myocardial injury (45, 46). Inflammation is associated with features of plaque instability, and an increased ratio of neutrophils to lymphocytes can predict adverse cardiovascular events (47, 48). As recently demonstrated, inflammation can contribute to the destabilization of atherosclerotic plaques and lead to future cardiovascular outcomes even in patients with myocardial infarction with non-obstructive coronary arteries (MINOCA) (49). Inhibiting inflammatory responses presents a promising medical intervention for AMI patients (50). This would explain why NLR and all-cause mortality were positively associated.

This is the initial study to confirm the link between NLR and all-cause mortality for AMI patients in the CCU. NLR is accessible and cost-effective. This study potentially provides valuable insights into the link between elevated NLR and increased all-cause in-hospital, 30-day, and 90-day mortality in AMI patients in the CCU, thereby helping CCU physicians identify AMI patients at high mortality risk. Stratification of individuals at high mortality risk can strengthen the communication and contact between physicians and patients' families about the patient's prognosis. It also allows CCU physicians to reduce MACEs through closer monitoring and more accurate early clinical decision-making. Some limitations also exist. Initially, retrospective bias is inevitable because this was a retrospective study. Secondly, our data came from the MIMIC-IV database in the United States, which mainly involves the white population. Caution should therefore be taken in interpreting the results for other races. Thirdly, given the limitations of MIMIC IV, some traditional inflammatory indicators and important confounding factors are seriously missing, AMI patients were screened based on the International Classification of Diseases, 9th and 10th revision. The 9th revision did not provide a precise classification of STEMI/NSTEMI patients. Thereby, more prospective studies are warranted to validate the correlation between NLR and all-cause mortality in AMI patients.

## Conclusion

NLR and all-cause mortality in AMI patients in the CCU are positively and nonlinearly correlated. Furthermore, NLR is superior to SII, PLR, and SIRI for predicting in-hospital, 30-day, and 90-day mortality rates.

## Data Availability

The original contributions presented in the study are included in the article/Supplementary Material, further inquiries can be directed to the corresponding authors.
