# Supplementary material for: Prognostic value of inflammatory markers for all-cause mortality in patients with acute myocardial infarction in the coronary care unit: a retrospective study based on MIMIC-IV database
Source: Front Cardiovasc Med. 2025 Jan 22;12:1439650. doi: 10.3389/fcvm.2025.1439650 (PMC11794309; doi:10.3389/fcvm.2025.1439650)
Supplement: Supplementary file 1 [file Table1.docx]

| **Supplementary Table 1**. Baseline characteristics of patients grouped by in-hospital mortality. | | | | |
| --- | --- | --- | --- | --- |
| Characteristic | Total(n=1386) | Suvival(n=1233） | Death(n=153） | p |
| Admission age (years) | 71.53 [62.70, 80.66] | 71.05 [62.17, 79.97] | 76.90 [68.05, 84.33] | <0.001 |
| Gender (male) | 827 (59.7%) | 743 (60.3%) | 84 (54.9%) | 0.235 |
| Race (other) | 300 (21.6%) | 286 (23.2%) | 14 (9.2%) | <0.001 |
| Race (white) | 885 (63.9%) | 794 (64.4%) | 91 (59.5%) |  |
| Race (unknow) | 201 (14.5%) | 153 (12.4%) | 48 (31.4%) |  |
| BMI (kg/m2) | 28.40 [24.60, 33.20] | 28.50 [24.60, 33.20] | 27.70 [24.50, 32.30] | 0.438 |
| CCI | 7.00 [5.00, 9.00] | 8.00 [5.00, 9.00] | 7.00 [6.00, 9.00] | 0.816 |
| Vital signs |  |  |  |  |
| SBP (mmHg) | 128.00 [117.00, 142.00] | 129.00 [117.00, 142.00] | 123.00 [112.00, 132.00] | 0.001 |
| DBP (mmHg) | 71.00 [62.00, 80.00] | 70.00 [62.00, 80.00] | 72.00 [62.00, 80.00] | 0.587 |
| Oxygen saturation (%) | 97.00 [95.00, 99.00] | 97.00 [95.00, 99.00] | 96.00 [93.00, 100.00] | 0.072 |
| Temperature (℃) | 36.67 [36.44, 36.89] | 36.67 [36.44, 36.89] | 36.56 [36.22, 36.89] | 0.003 |
| Laboratory parameters |  |  |  |  |
| Hemoglobin (g/dL) | 12.70 [11.10, 14.07] | 12.80 [11.20, 14.10] | 12.00 [10.60, 13.50] | <0.001 |
| Hemoglobina1c (%) | 6.00 [5.60, 7.00] | 6.00 [5.60, 7.00] | 6.00 [5.60, 7.00] | 0.79 |
| Total bilirubin (mg/dL) | 0.50 [0.30, 0.80] | 0.50 [0.30, 0.80] | 0.60 [0.40, 1.00] | 0.002 |
| INR | 1.10 [1.00, 1.30] | 1.10 [1.00, 1.20] | 1.20 [1.10, 1.60] | <0.001 |
| BUN (mmol/L) | 21.00 [16.00, 30.00] | 20.00 [15.00, 28.00] | 25.00 [18.00, 40.00] | <0.001 |
| Creatinine (mg/dL) | 1.10 [0.90, 1.40] | 1.00 [0.80, 1.40] | 1.30 [1.00, 1.70] | <0.001 |
| Platelet count(109/L) | 228.00 [179.00, 282.00] | 229.00 [183.00, 282.00] | 208.00 [166.00, 280.00] | 0.012 |
| Lymphocyte count(109/L) | 1.35 [0.89, 1.98] | 1.37 [0.92, 2.01] | 1.12 [0.72, 1.76] | 0.005 |
| Neutrophil count(109/L) | 6.86 [4.73, 10.44] | 6.62 [4.66, 9.95] | 10.06 [6.54, 15.07] | <0.001 |
| Monocyte count(109/L) | 0.72 [0.52, 0.97] | 0.71 [0.52, 0.94] | 0.82 [0.55, 1.09] | 0.011 |
| Clinical therapy , n (%) |  |  |  |  |
| Aspirin | 1327 (95.7%) | 1195 (96.9%) | 132 (86.3%) | <0.001 |
| Clopidogrel | 845 (61.0%) | 775 (62.9%) | 70 (45.8%) | <0.001 |
| Heparin | 1355 (97.8%) | 1207 (97.9%) | 148 (96.7%) | 0.532 |
| Tirofiban | 73 (5.3%) | 67 (5.4%) | 6 (3.9%) | 0.55 |
| Statin | 1303 (94.0%) | 1182 (95.9%) | 121 (79.1%) | <0.001 |
| Esmolol | 39 (2.8%) | 34 (2.8%) | 5 (3.3%) | 0.92 |
| Amiodarone | 410 (29.6%) | 356 (28.9%) | 54 (35.3%) | 0.122 |
| Digoxin | 128 (9.2%) | 119 (9.7%) | 9 (5.9%) | 0.17 |
| Milrinone | 87 (6.3%) | 76 (6.2%) | 11 (7.2%) | 0.751 |
| PCI | 285 (20.6%) | 281 (22.8%) | 4 (2.6%) | <0.001 |
| CABG | 243 (17.5%) | 235 (19.1%) | 8 (5.2%) | <0.001 |
| Comorbidities, n (%) |  |  |  |  |
| diabetes | 691 (49.9%) | 626 (50.8%) | 65 (42.5%) | 0.065 |
| Cerebral infarction | 139 (10.0%) | 124 (10.1%) | 15 (9.8%) | 1 |
| COPD | 275 (19.8%) | 249 (20.2%) | 26 (17.0%) | 0.407 |
| Liver disease | 74 (5.3%) | 67 (5.4%) | 7 (4.6%) | 0.799 |
| Renal disease | 152 (11.0%) | 142 (11.5%) | 10 (6.5%) | 0.085 |
| Scoring systems |  |  |  |  |
| APS III | 48.00 [35.00, 63.00] | 46.00 [33.00, 60.00] | 66.00 [55.00, 83.00] | <0.001 |
| OASIS | 33.00 [27.00, 40.00] | 32.00 [26.00, 39.00] | 40.00 [34.00, 47.00] | <0.001 |
| SIRS | 3.00 [2.00, 3.00] | 3.00 [2.00, 3.00] | 3.00 [2.00, 4.00] | <0.001 |
| SAPS II | 40.00 [31.00, 51.00] | 39.00 [30.00, 49.00] | 51.00 [44.00, 65.00] | <0.001 |
| GCS | 14.00 [11.00, 15.00] | 14.00 [12.00, 15.00] | 12.00 [7.00, 15.00] | <0.001 |
| LODS | 3.00 [1.00, 5.00] | 3.00 [1.00, 4.00] | 7.00 [5.00, 10.00] | <0.001 |
| BMI, body mass index; CCI, charlson comorbidity index; SBP, systolic blood pressure; DBP, diastolic blood pressure; INR, international normalized ratio; BUN, blood urea nitrogen; PCI, percutaneous coronary intervention; CABG, coronary artery bypass grafting; COPD, chronic obstructive pulmonary disease; APS III, acute physiology score III; OASIS, oxford acute severity of illness score; SIRS, systemic inflammatory response syndrome score; SAPS II, simplified acute physiology score II; GCS, glasgow coma scale; LODS, logistic organ dysfunction system. | | | | |
